# Exploring the Distinct Regulatory Actions and Molecular Pathways of Coptisine Through Network Pharmacology: Insights Into Its Influence on Platelet Activation and Thrombus Development

**DOI:** 10.1002/fsn3.72107

**Published:** 2026-07-15

**Authors:** Yanli Liu, Ming Qian, Hui Yang, Shiyu Qian, Depin Li, Xiaoli Zhou, Xian Yang, Yihai Liu

**Affiliations:** ^1^ Department of Pharmacy, Nanjing Drum Tower Hospital, Affiliated Hospital of Medical School Nanjing University Nanjing China; ^2^ Health College Yuncheng Vocational and Technical University Yuncheng Shanxi China; ^3^ Department of Cardiology, Nanjing Drum Tower Hospital, Affiliated Hospital of Medical School Nanjing University Nanjing China; ^4^ State Key Laboratory of Functions and Applications of Medicinal Plants Guizhou Medical University Guiyang China

**Keywords:** coptisine, molecular docking, network pharmacology, platelet activation, thrombosis

## Abstract

To investigate the potential targets and underlying mechanisms of coptisine in regulating platelet activation and thrombosis by integrating an approach combining network pharmacology and experimental validation. An in vivo carotid artery thrombosis model induced by FeCl_3_‐mediated endothelial injury was established in mice to evaluate the antithrombotic effects of coptisine. A combination of network pharmacology prediction and both in vivo and in vitro experimental validation was employed to elucidate the antithrombotic mechanisms of coptisine. Molecular docking simulations were performed using AutoDock Vina to assess the binding interactions between coptisine and its targets. The in vitro antiplatelet effects of coptisine were assessed using platelet aggregation assays induced by thrombin, ADP, and collagen, as well as a thrombin‐induced platelet activation model. Administration of coptisine significantly reduced thrombus formation in the mouse model of carotid artery thrombosis. Network pharmacology analysis identified four key genes—NFKB1, PTGS2, STAT1, and ACE—as potential core targets contributing to the antithrombotic effects of coptisine. Functional enrichment analysis further indicated the involvement of these targets in pathways relevant to thrombosis and inflammation. Molecular docking studies demonstrated strong binding affinities between coptisine and the identified targets. Both in vivo and in vitro studies confirmed that coptisine treatment markedly attenuated thrombin‐induced platelet activation, as evidenced by reduced platelet inflammation and decreased levels of platelet granule release. This study demonstrates that coptisine exerts significant protective effects against thrombosis‐related diseases through modulation of the NF‐κB and MAPK signaling pathways.

## Graphical abstract

The methodological framework of this study integrates computational network pharmacology analysis with subsequent experimental validation.

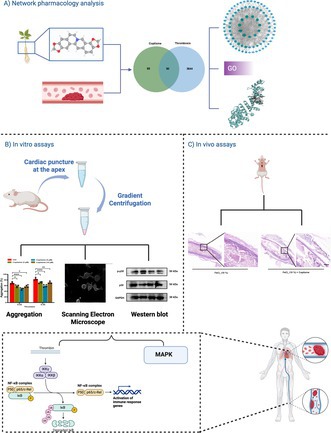

**Abbreviations**
ADP: adenosine diphosphate
ATP: adenosine triphosphate
IL‐1β: interleukin 1 beta
IL‐6: interleukin 6
KEGG: Kyoto Encyclopaedia of Genes and Genomes
NFKB1: nuclear factor kappa B subunit 1
NF‐κB: nuclear factor kappa B
PPP: platelet poor plasma
PRP: platelet rich plasma
TCMSP: traditional Chinese medicine systems pharmacology database and analysis platform
TEM: transmission electron microscopy
TNF‐α: tumor necrosis factor alpha
TXA2: thromboxane A2

## 1 Introduction

Thrombotic disorders, such as myocardial infarction, ischemic stroke, and venous thromboembolism, all share a common pathological basis: thrombosis (Abbassi et al. 2025). Despite the widespread clinical use of antiplatelet agents (e.g., aspirin and clopidogrel), approximately 30% of patients still exhibit “therapeutic thrombosis resistance” (Patrono and Rocca 2022). Aberrant platelet activation is a crucial event in thrombus formation. Activated platelets not only promote intravascular thrombosis but also directly contribute to cerebral artery occlusion, thereby triggering stroke (Thomas and Storey 2015). This process involves complex intracellular signaling cascades, including cytoskeletal reorganization, release of α‐granules and dense granules, and conformational changes in integrin adhesion receptors (particularly αIIbβ3), which lead to altered affinity states. These interconnected biological events collectively drive the progression of thrombotic diseases (Yeung et al. 2018). Targeted modulation of platelet activation is a key strategy for preventing and treating thrombotic disorders. Thus, deeper insight into its molecular mechanisms is essential for developing more effective antithrombotic therapies and improving clinical outcomes.

In recent years, Traditional Chinese Medicine (TCM) has demonstrated significant clinical value in the treatment of cardiovascular diseases (Petraina et al. 2022). As a classic TCM herb, Rhizoma Coptidis (Coptis chinensis) contains one of its major active components, Coptisine (C19H14NO4, see Figure 4A for chemical structure), which has garnered considerable attention due to its pleiotropic pharmacological effects (Wu, Luo, Deng, et al. 2019). Existing studies have shown that Coptisine possesses multiple activities, including anti‐inflammatory, antioxidant, cardioprotective, and nephroprotective properties (Lu et al. 2023; Nie et al. 2024). Research has indicated that Coptisine exerts therapeutic effects on gouty arthritis by inhibiting the activation of the NLRP3 inflammasome and caspase‐1 (Wu, Luo, Jiang, et al. 2019). Additionally, Coptisine has been shown to exhibit anti‐atherosclerotic and anti‐myocardial ischemia effects through mechanisms involving AMPK activation‐mediated lipid metabolism improvement and regulation of oxidative stress and cell apoptosis via p38 MAPK inhibition, drawing attention for its anti‐inflammatory, antioxidant, and cholesterol‐lowering properties (Tao et al. 2025). Therefore, Coptisine holds significant importance in cardiovascular diseases and inflammation‐related disorders.

Inflammation plays a crucial role in regulating platelet function through multiple mechanisms. Pro‐inflammatory cytokines (e.g., TNF‐α and IL‐6) directly activate platelets, enhancing their aggregation and adhesion (Badraoui et al. 2025). Activated platelets release mediators such as CD40L and IL‐1β, which recruit white blood cells and amplify inflammation. The positive feedback loop formed among platelets, white blood cells, and neutrophil extracellular traps (NETs) further exacerbates thromboinflammatory responses (Thakur et al. 2023). Given the central role of inflammation in platelet activation and thrombosis, we integrated in vivo and in vitro pharmacological experiments with network pharmacology to verify that coptisine disrupts the “inflammation–platelet–thrombus” cycle by inhibiting platelet‐mediated inflammatory responses, offering a novel and effective strategy for treating thrombotic disorders.

In this study, we combined network pharmacology with experimental validation to explore the antithrombotic potential of coptisine. PPI network analysis identified its protein targets, and molecular docking predicted binding affinities, revealing mechanistic insights into thrombosis treatment. The effects of coptisine on thrombin‐induced platelet function were assessed at cellular and organismal levels using Western blotting, immunohistochemistry, and flow cytometry in both in vitro and ex vivo settings. The overall research workflow is shown in the Graphical Abstract.

## 2 Materials and Methods

### 2.1 Chemicals and Reagents

Coptisine (HY‐N0430) was purchased from MCE (Shanghai, China). Protein quantification was performed with a bicinchoninic acid (BCA) protein detection kit (P0012, Beyotime Institute of Biotechnology, Shanghai, China). For immunoblotting analysis, rabbit‐derived primary antibodies targeting phosphorylated p38 MAPK and total p38 MAPK were procured from Cell Signaling Technology (Massachusetts, USA), with both antibodies being applied at a 1:1000 dilution. Corresponding secondary antibodies included horseradish peroxidase‐conjugated goat anti‐rabbit IgG (A0208) and goat anti‐mouse IgG (A0216), supplied by Beyotime Institute of Biotechnology (Shanghai, China). Flow cytometry reagents consisting of fluorescein isothiocyanate‐labeled integrin αIIbβ3 (362803) and phycoerythrin‐conjugated CD62P (148036) were acquired from BioLegend (California, USA). Essential coagulation components including fibrinogen (341576) and thrombin (T7513) were sourced from Sigma‐Aldrich (Missouri, USA).

### 2.2 Data Collection

The chemical profile of coptisine was extracted from the TCMSP database (https://tcmsp‐e.com/). Comprehensive molecular characterization, including standardized nomenclature, PubChem compound identifiers (CID), and spatial conformation data, was derived from the PubChem database (https://pubchem.ncbi.nlm.nih.gov/). Putative biological targets were predicted using the PharmMapper computational platform. Thrombosis‐associated molecular targets were systematically collected from the GeneCards human gene database (https://www.genecards.org/; Li, Chen, et al. 2024). Intersection analysis between coptisine's potential targets and thrombosis‐related targets was performed employing Venny software (version 2.1.0) to determine overlapping molecular targets of therapeutic relevance.

### 2.3 Protein–Protein Interaction (PPI) Analyses

All network analyses were performed using Cytoscape software (version 3.8.1). First, common targets were analyzed using the STRING database (https://cn.string‐db.org/; Li, Xin, et al. 2024) with a confidence score threshold set to > 0.4 to construct a protein–protein interaction (PPI) network. In the constructed PPI network, targets are represented as nodes, and interactions between targets are represented as edges. The network comprised 88 nodes and 818 edges. In the analysis results, the color and size of the nodes represent their degree values: darker colors and larger nodes indicate higher degree values and greater target importance.

### 2.4 Enrichment Analysis and Network Construction

Functional annotation and pathway analysis were conducted for the identified common targets and hub genes using the KOBAS 3.0 bioinformatics platform. Statistical significance was established at *p* values below 0.05 for all enrichment results. The most relevant GO (top 10 ranked entries by *p* value) terms and KEGG (top 20 ranked entries by *p* value) were graphically represented through multiple visualization formats (Zhang et al. 2024). Susquently, the top five genes from the entire PPI network were selected as the most critical hub genes. The STRING database revealed interactions among these five targets. Finaly, GO and KEGG pathway enrichment analyses were performed on these five targets. All results were filtered at a significance level of *p* < 0.05. The analysis results were visualized as bar plots and bubble charts using the bioinformatics online platform (http://www.bioinformatics.com.cn/).

The “drug‐component‐target” network was visualized using Cytoscape software (version 3.7.1). The CytoHubba plugin was applied to analyze node degree values, and hub genes were identified based on the median degree value.

### 2.5 Molecular Docking

The target protein information was retrieved from the UniProt database (https://www.uniprot.org/), and their structural files in PDB format were obtained from the RCSB Protein Data Bank, including NFKB1 (PDB ID: 7RG5), PTGS2 (PDB ID: 5F19), STAT1 (PDB ID: 3WWT), and ACE (PDB ID: 6H5W). The corresponding three‐dimensional chemical structures of the compounds were collected from the PubChem database or drawn using ChemDraw software. Subsequently, AutoDockTools‐1.5.7 was employed to preprocess the protein structures, including the removal of water molecules, addition of hydrogen atoms, and calculation of charge distribution. Meanwhile, the compound molecules were preprocessed by adding hydrogen atoms and setting rotatable bonds. Then, molecular docking simulations were performed using AutoDock Vina to calculate the binding affinities.

For the target proteins NFKB1 (PDB ID: 7RG5), PTGS2 (PDB ID: 5F19), STAT1 (PDB ID: 3WWT), and ACE (PDB ID: 6H5W), the molecular docking grid parameters were set as follows: For NFKB1, the grid center coordinates were set to *x* = −13.344, *y* = 6.275, *z* = −21.94, and the grid box dimensions were set to *x* = 71.88, *y* = 74.41, *z* = 79.45; For PTGS2, the grid center coordinates were set to *x* = 22.595, *y* = 40.994, *z* = 39.573, and the grid box dimensions were set to *x* = 80.383, *y* = 77.35, *z* = 95.55; For STAT1, the grid center coordinates were set to *x* = −29.045, *y* = 7.957, *z* = −12.596, and the grid box dimensions were set to *x* = 55.5, *y* = 45.222, *z* = 64.75; For ACE, the grid center coordinates were set to *x* = −12.15, *y* = −5.401, *z* = 24.508, and the grid box dimensions were set to *x* = 64.122, *y* = 69.65, *z* = 69.65. The grid spacing was set to the default value of 1.0 for all docking calculations, and the number of docking modes was set to 9 for all. Each protein‐ligand pair was subjected to nine independent docking calculations, and the conformation with the lowest binding energy was selected for subsequent analysis; the exhaustiveness parameter for the docking process was kept at its default value. Finally, PyMOL 2.6.0 software was used for three‐dimensional visualization and analysis of the docking results (Badraoui et al. 2025).

### 2.6 Experimental Animals

Ethical approval for the entire study was granted by the Ethics Review Committee of Nanjing Drum Tower Hospital, Nanjing University (approval number: DWSY‐25070702). For the experiments, we obtained male C57BL/6 mice (6–8 weeks old, body weight 23–25 g) from SPF Biotechnology Co. (Nanjing, China). In this study, only male mice were used to avoid the potential confounding effects of hormonal cycles on platelet function (Butkiewicz et al. 2006; Coleman et al. 2019; Leng et al. 2004; Li, Zhang, et al. 2024; Zhang et al. 2023). We acknowledge that the exclusion of female mice constitutes a limitation, as the role of coptisine in platelets from female mice was not investigated. Future studies should include both sexes to assess potential sex‐specific responses.

For the in vivo evaluation of coagulation function, mice were randomly divided into three groups (*n* = 5): (1) vehicle control group (0.9% saline, i.g.), (2) coptisine low‐dose group (10 mg/kg, i.g.), and (3) coptisine high‐dose group (30 mg/kg, i.g.). Drug or vehicle was administered once daily for 7 consecutive days prior to thrombosis induction. For the antithrombotic efficacy evaluation, mice were randomly divided into three groups (*n* = 3): (1) FeCl_3_‐induced carotid artery injury without treatment (2) FeCl_3_‐induced carotid artery injury with coptisine treatment (30 mg/kg, i.g.), and (3) FeCl_3_‐induced carotid artery injury with aspirin treatment (30 mg/kg, i.g.).

For ex vivo platelet aggregation assays, platelets were divided into experimental control, coptisine‐treated (2, 5, and 10 μM), and aspirin‐treated positive control groups, and stimulated with thrombin, ADP, or collagen (*n* = 4). For αIIbβ3 activation (JON/A binding), α‐granule secretion (P‐selectin), and ATP release, groups included resting control, agonist, coptisine‐treated (2.5 and 5 μM), and aspirin‐treated positive control (*n* = 5). For platelet adhesion, protein expression, and inflammatory cytokines (IL‐1β, IL‐6, TNF‐α), groups included resting control, agonist, and coptisine‐treated (2.5 and 5 μM) (*n* = 4–5). Animal numbers were determined based on established practices in previous similar studies and practical considerations (Liu, Zhao, et al. 2025; Qi et al. 2021; Xin et al. 2017). Prior to experimentation, all animals were housed for seven days in a controlled‐environment facility meeting specific pathogen‐free standards, where ambient conditions were carefully maintained at 20°C–22°C with 55% relative humidity under standardized 12‐h photoperiod conditions (light/dark cycle).

### 2.7 Platelet Preparation

Blood was collected from pentobarbital‐narcotized mice from the heart into 3.8% citrate anticoagulant (Xin et al. 2017). Platelet isolation was achieved through a two‐step centrifugation protocol: initial centrifugation at 250 *g* for 6 min to obtain platelet‐rich plasma (PRP), followed by a second centrifugation at 600 *g* for 5 min to pellet platelets. The isolated platelets were then resuspended in a physiological Tyrode's solution (composition in mM: 20 HEPES, 137 sodium chloride, 13.8 sodium bicarbonate, 2.5 potassium chloride, 0.36 sodium phosphate monobasic, and 5.5 glucose; pH adjusted to 7.4) to achieve the desired platelet concentration.

### 2.8 Platelet Aggregation

Platelet aggregation was analyzed using a platelet aggregometer (AG400) at 37°C under stirring conditions (1200 rpm) for 5 min, with thrombin (0.025 U/mL) used to stimulate washed platelets (2 × 10^8^/mL) from both normal and Coptisine‐treated mice. Experiments were conducted after normalizing the platelet count to 2 × 10^8^/mL. Platelets were incubated with Coptisine for 30 min prior to the experiment (Liu, Li, et al. 2025).

### 2.9 Scanning Electron Microscopy Analysis

Washed platelets were fixed in 3% glutaraldehyde in 0.1 M sodium carbonate buffer (pH 7.4) and dehydrated with gradient ethanol. The samples were placed on 2 mm glass slides, sputtered with gold, and dried (Gauer et al. 2023). The specimens were observed under a ZEISS EVO10 scanning electron microscope.

### 2.10 Platelet Adhesion

The experimental procedure involved seeding platelet suspensions (2 × 10^7^ cells/mL) onto fibrinogen‐precoated glass coverslips (prepared by overnight incubation with 100 μg/mL fibrinogen at 4°C), followed by a 90‐min adhesion period at physiological temperature (37°C). Non‐adherent cells were removed by gentle washing with HEPES‐buffered Tyrode's solution. Subsequently, adherent platelets were subjected to fixation and permeabilization treatments before being labeled with Alexa Fluor 546‐conjugated phalloidin for F‐actin visualization. Fluorescence imaging was conducted using a Nikon Eclipse 80i microscope equipped with a 100× oil‐immersion objective lens for high‐resolution observation (Manikanta et al. 2025).

### 2.11 ATP Secretion Detection

After washing, 100 μL of Cell‐Titer‐Lumi Steady Luminescence Detection Solution was added to each well containing the platelets, and the luminescence intensity was measured using a microplate luminometer (Liu, Wang, et al. 2023).

### 2.12 Flow Cytometry

The expression of P‐selectin (CD62P‐PE) and the active conformation of αIIbβ3 integrin (detected by JON/A binding) were measured using fluorescently labeled antibodies. The samples were incubated in the dark at room temperature for 20 min. Resting and washed platelets (at a concentration of 1 × 10^7^/mL) were activated with thrombin (0.01 U/mL) for 5 min and pretreated with Coptisine for 30 min. Data were analyzed using FlowJo v10 software.

### 2.13 Western Blot Analysis

Wild‐type (WT) washed platelets (250 μL, 2 × 10^8^/mL) were stimulated with thrombin in the presence or absence of Coptisine. Total proteins were extracted from platelets using RIPA lysis buffer. The proteins were separated by SDS‐PAGE gel electrophoresis and transferred onto PVDF membranes. The membranes were then incubated with primary antibodies against p‐P50 and P50 (1:1000; HUABIO) and GAPDH (1:1000; Abcam). After incubation with the corresponding secondary antibodies, the protein bands were visualized by chemiluminescence and quantified using the Odyssey Fc system (LI‐COR Biosciences) and Image Lab software (Liu, Zhang, et al. 2025).

### 2.14 FeCl3‐Induced Thrombosis Model

Mice were randomly divided into the following groups: control group, model group, and Coptisine groups (10, 30 mg/kg, i.g.). Based on previous literature reports and our preliminary experimental results, 30 mg/kg of coptisine exhibited optimal antithrombotic efficacy, with no significant additional benefit observed at higher doses. Based on the cumulative evidence, 30 mg/kg was selected as the optimal dose of coptisine in the present study (He et al. 2015; Liu et al. 2022; Luo et al. 2018; Qi et al. 2025; Tan et al. 2025b). After 1 week of administration, mice were anesthetized with 1% pentobarbital, and the carotid artery was carefully exposed and kept moist with saline. A piece of filter paper (5 × 3 mm) soaked in 10% ferric chloride (FeCl_3_) solution was applied to the carotid artery for 1 min to induce thrombosis (Liu, Zhao, et al. 2025).

### 2.15 Hematoxylin and Eosin (H&E) Staining

Thrombus samples were fixed and embedded in paraffin. Sections of the thrombi were stained with hematoxylin and eosin (H&E; Du et al. 2024). Images of the immunofluorescence staining were captured using an Axio Observer Z1 inverted fluorescence microscope. The images were processed using Zen 2012 (blue edition, version 2.3; Zeiss) software.

### 2.16 Statistical Analysis

All data are presented as mean ± standard deviation (SD). Statistical analyses were performed using GraphPad Prism 8.0 software. Prior to conducting parametric tests (including Student's *t*‐test and one‐way/two‐way ANOVA), the data were assessed for normality (e.g., Shapiro–Wilk test) and homogeneity of variance (e.g., Levene's test) to confirm compliance with the assumptions of parametric tests. In cases where these assumptions were not met, nonparametric tests were used instead. For analyses involving multiple comparisons, the Bonferroni method was applied to adjust the significance level. The threshold for statistical significance was set at *p* < 0.05. The sample size of animals was determined based on standard practices in the field, and post hoc power analysis confirmed that the study had sufficient statistical power (power > 0.8).

## 3 Results

### 3.1 Network Pharmacology Analysis Reveals the Potential Protective Effects of Coptisine Against Thrombosis

**FIGURE 1:**
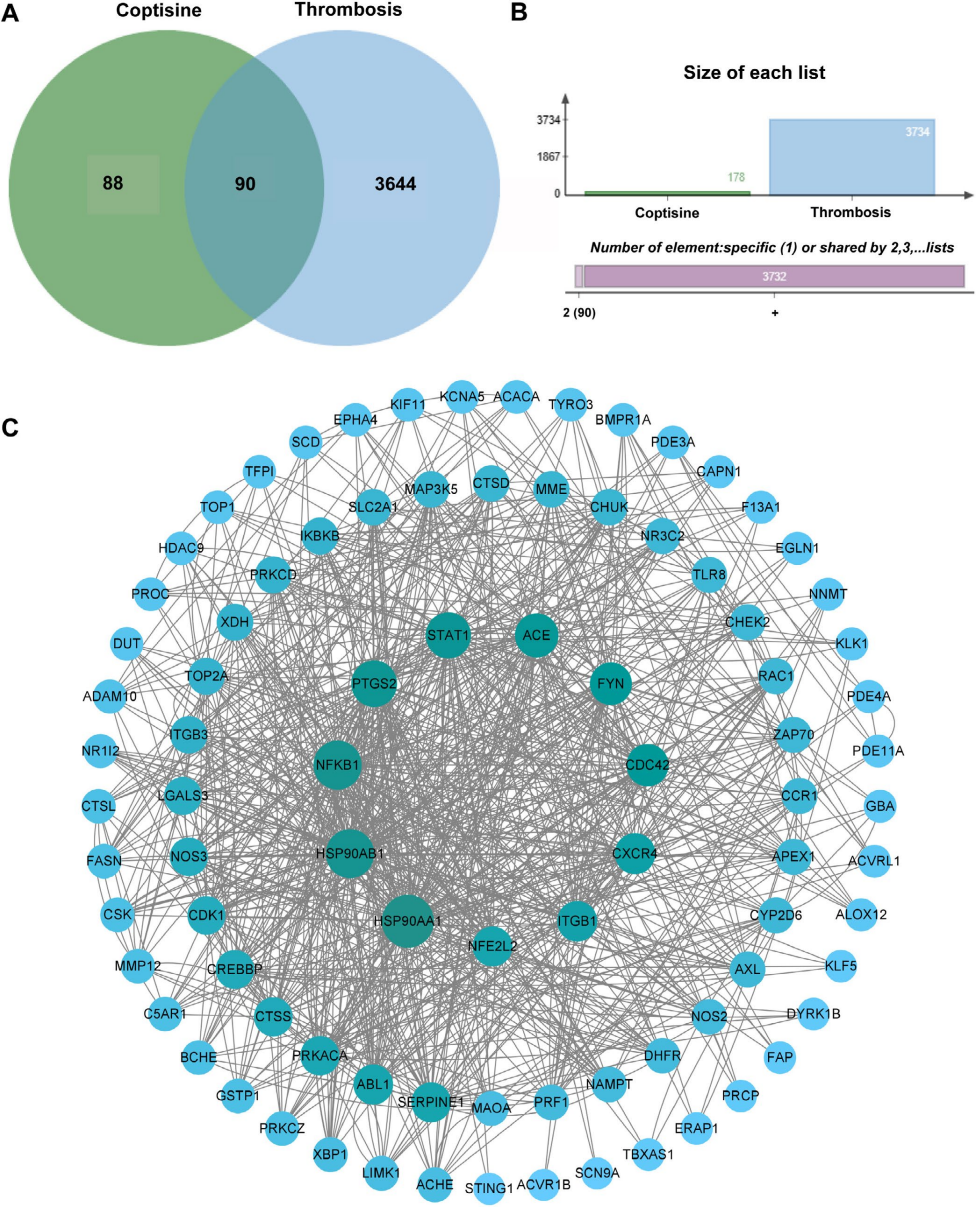
Network analysis of predicted targets of Coptisine in thrombosis. (A, B) Venn diagrams showing 90 targets, which are the intersection of the predicted targets of Coptisine and thrombosis‐related targets; (C) PPI network of the 90 common targets.

Venny analysis identified 90 common targets between compound and disease targets (3644 disease‐related targets; Figure 1A,B), representing the potential therapeutic targets of coptisine for thrombosis (Figure 1C). Among them, NFKB1 was found to regulate key pro‐inflammatory cytokines (e.g., TNFα, IL‐1, IL‐6, IL‐12), chemokines (e.g., CXCL1, CXCL2, RANTES), and adhesion molecules (e.g., VCAM‐1) involved in inflammation‐regulating cell activation and recruitment.

### 3.2 Target Biological Function Analysis

**FIGURE 2:**
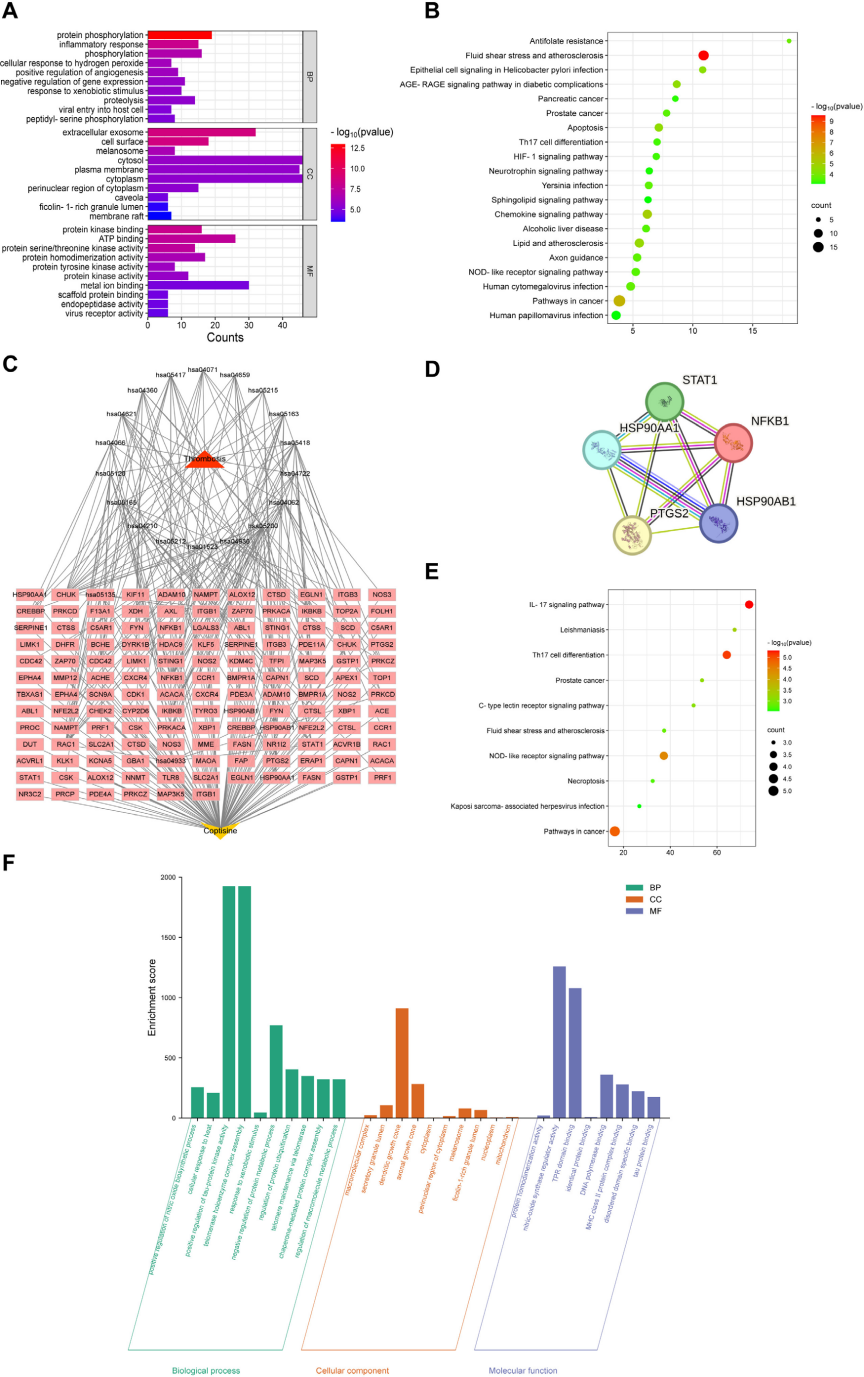
Enrichment analysis results of core targets of Coptisine against thrombosis. (A) Top 10 GO enrichment entries for the core targets of Coptisine against thrombosis (BP, biological process; CC, cellular component; MF, molecular function), (B) Top 20 KEGG pathway enrichment entries for the core targets of Coptisine against thrombosis, (C) Interaction network diagram of Coptisine‐thrombosis‐targets, (D) Protein–protein interaction network of the top five core targets, (E) KEGG pathway enrichment analysis of the five core targets (top 10 significant pathways), (F) Top 10 GO analysis of the 5 hub targets.

GO and KEGG analyses of the 90 targets showed enrichments in cytokine signaling, protein phosphorylation, inflammatory response regulation (BP); extracellular vesicles, cytosol, cytoplasm, nucleus (CC); protein/enzyme/serine/threonine kinase binding (MF), and notably, platelet activation pathways (Figure 2A,B). Hub node analysis identified five key targets enriched in IL‐17 signaling and necroptosis pathways—both critical for platelet function (Figure 2C–F). Collectively, these results suggest that coptisine exerts antithrombotic effects by inhibiting platelet inflammation, thereby affecting platelet activation, adhesion, and spreading, with the NF‐κB pathway as a likely key mediator.

### 3.3 Molecular Docking Identifies Potential Anti‐Thrombosis Active Compounds

**FIGURE 3:**
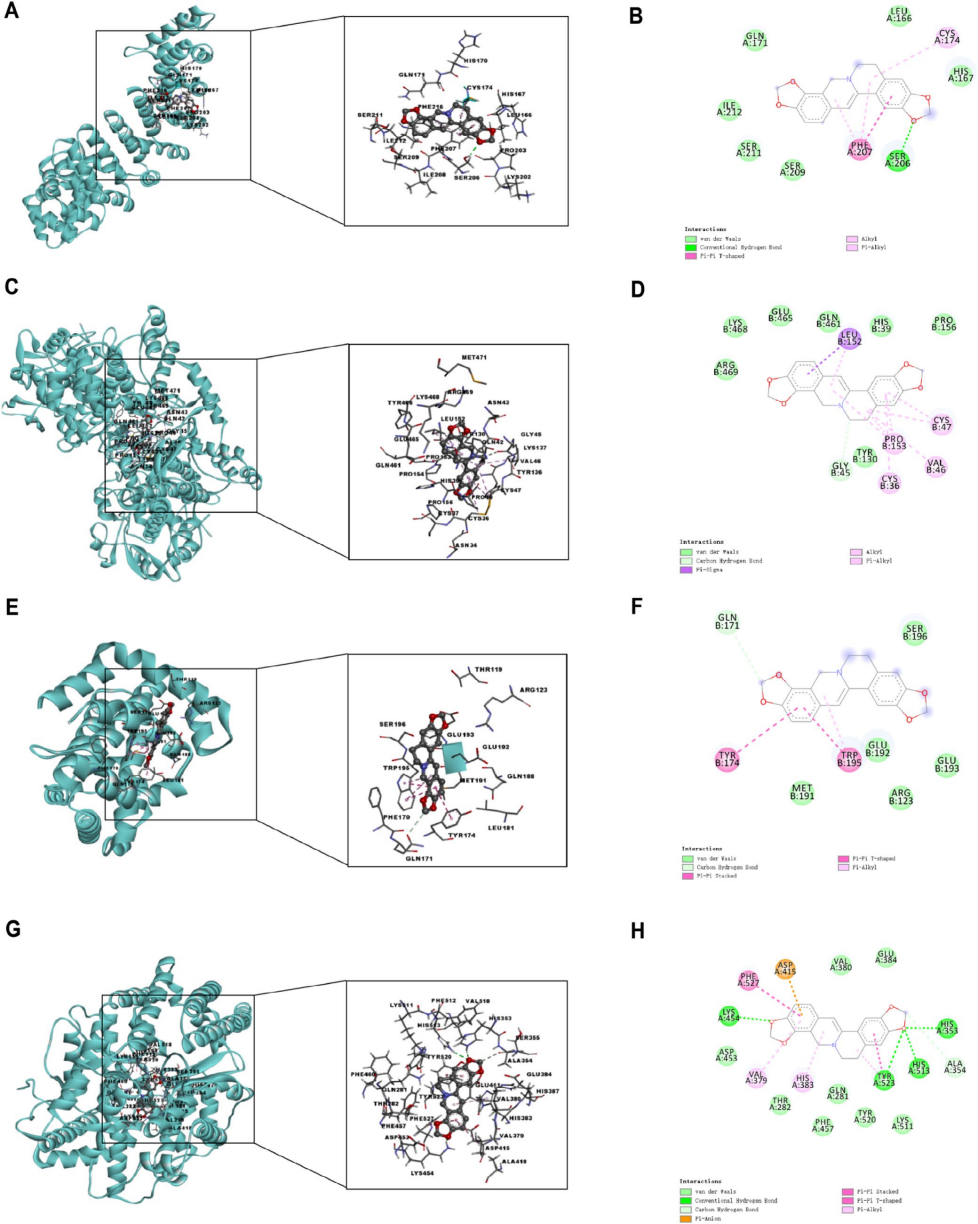
Molecular docking results and 2D docking diagrams of Coptisine (A, B) Docking results for NFKB1, (C, D) PTGS2, (E, F) STAT1, and (G, H) ACE.

Next, we validated the interactions between Coptisine and its potential targets through molecular docking analysis. A lower docking score is positively correlated with stronger ligand‐receptor binding affinity, indicating a higher likelihood of interaction. The binding energies of all targets with Coptisine were below −5.0 kcal/mol, with NFKB1 exhibiting a binding energy as low as −8.3 kcal/mol, suggesting that it may be a key target for Coptisine in the treatment of thrombosis (Figure 3A–H).

### 3.4 Coptisine Inhibit Platelet Activation In Vitro

**FIGURE 4:**
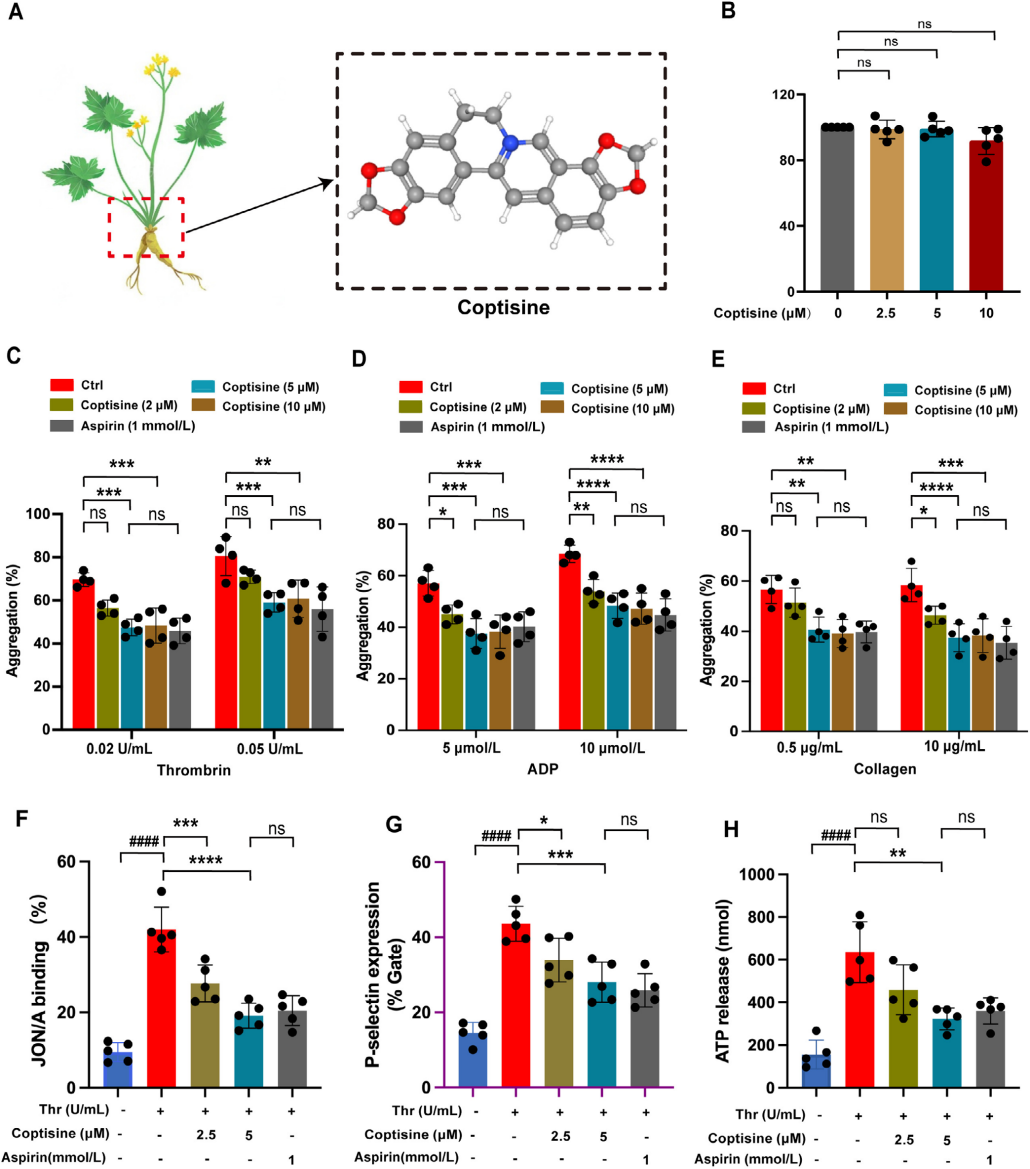
Coptisine inhibits agonist‐induced platelet activation in vitro. (A) Chemical structure of Coptisine. (B) Effects of Coptisine on platelet viability. *n* = 5, ns = *p* > 0.05. (C, D, E) Effects of Coptisine on platelet aggregation induced by ADP, thrombin, or collagen. Data are expressed as mean ± SD. *n* = 4. *p < 0.05, **p < 0.01, ***p < 0.001 ns = *p* > 0.05. (F, G) Flow cytometric analysis of PE‐JON/A and FITC‐CD62P binding to platelets after stimulation with thrombin for 5 min at 37°C. Data are expressed as mean ± SD. *n* = 5. ###*p* < 0.001 versus the control, *p < 0.05, **p < 0.01, ***p < 0.001 versus the model group. ns = *p* > 0.05. (H) Effects of Coptisine on platelet ATP secretion. Data are expressed as mean ± SD. *n* = 5. ###*p* < 0.001 versus the control, *p < 0.05, **p < 0.01, ***p < 0.001 versus the model group. ns = *p* > 0.05.

Coptisine, a plant alkaloid with anti‐inflammatory properties (Figure 4A), was identified via network pharmacology as a potential key compound for thrombotic inflammation. In vitro validation showed no significant cytotoxicity to platelets (Figure 4B). Turbidimetry revealed that 2–10 μM coptisine inhibited platelet aggregation induced by thrombin, ADP, and collagen (Figure 4C–E), with 5 μM coptisine comparable to aspirin (1 mmol/L). Coptisine (5 μM) also reduced thrombin‐induced integrin activation (JON/A binding) and P‐selectin release (Figure 4F,G), as well as ATP release (Figure 4H), similar to aspirin. This effective concentration is consistent with the concentration ranges used in previous studies involving coptisine (Yu et al. 2015). These results demonstrate that coptisine effectively inhibits agonist‐induced platelet aggregation and dense granule release.

### 3.5 Coptisine Inhibits Platelet Adhesion

**FIGURE 5:**
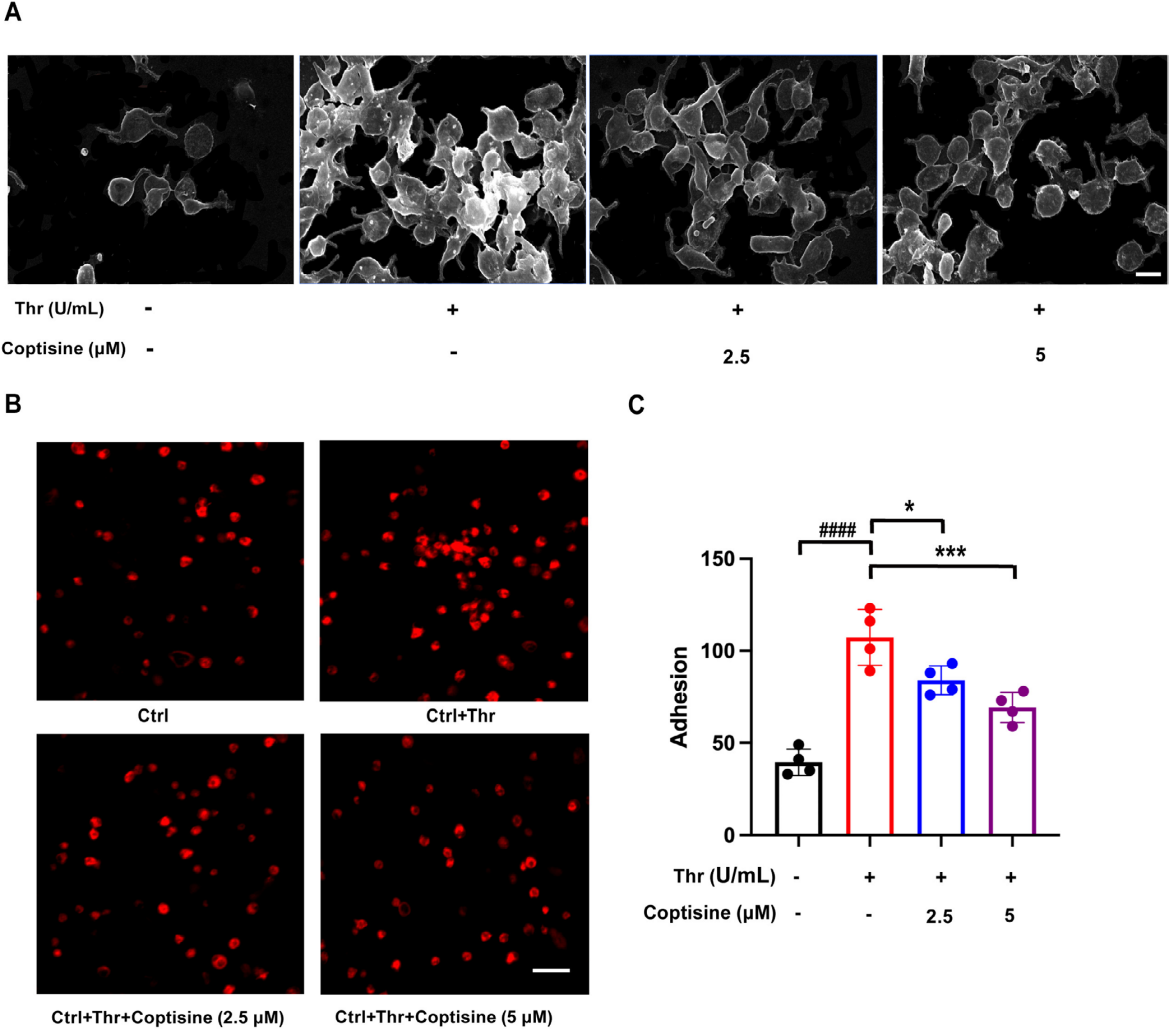
Coptisine inhibits agonist‐induced platelet activation and adhesion (A) Scanning electron microscopy was used to observe the effects of Coptisine on platelet morphology, scale bar represents 5 μm, *n* = 3. (B) Effects of Coptisine on thrombin‐induced platelet adhesion; scale bar represents 10 μm. (C) Platelet numbers were measured using ImageJ software. Data are expressed as mean ± standard deviation. *n* = 4. ###*p* < 0.001, versus the control group, *p < 0.01, **p < 0.01, ***p < 0.001 versus the model group.

The adhesion and spreading of platelets at sites of vascular injury are critical steps in hemostasis and thrombus formation (van der Meijden and Heemskerk 2019). Scanning electron microscopy revealed that after thrombin stimulation, Coptisine differentially regulated the morphology of platelets and intercellular connections. In the low‐concentration thrombin (0.01 U/mL) stimulation group, platelet pseudopod formation was significant, while the number of pseudopods in platelets treated with Coptisine was reduced, with the most pronounced effect observed in the 5 μM Coptisine group (Figure 5A). Subsequent platelet adhesion experiments targeting fibrinogen confirmed that after thrombin stimulation, the number of adherent platelets in the thrombin control group significantly increased, while Coptisine significantly inhibited platelet adhesion, suggesting its ability to inhibit the platelet adhesion/spreading pathway (Figure 5B,C).

### 3.6 Coptisine Regulates Cellular Inflammation and Inhibits Platelet Activation via the NF‐κB/p38‐MAPK Pathway

**FIGURE 6:**
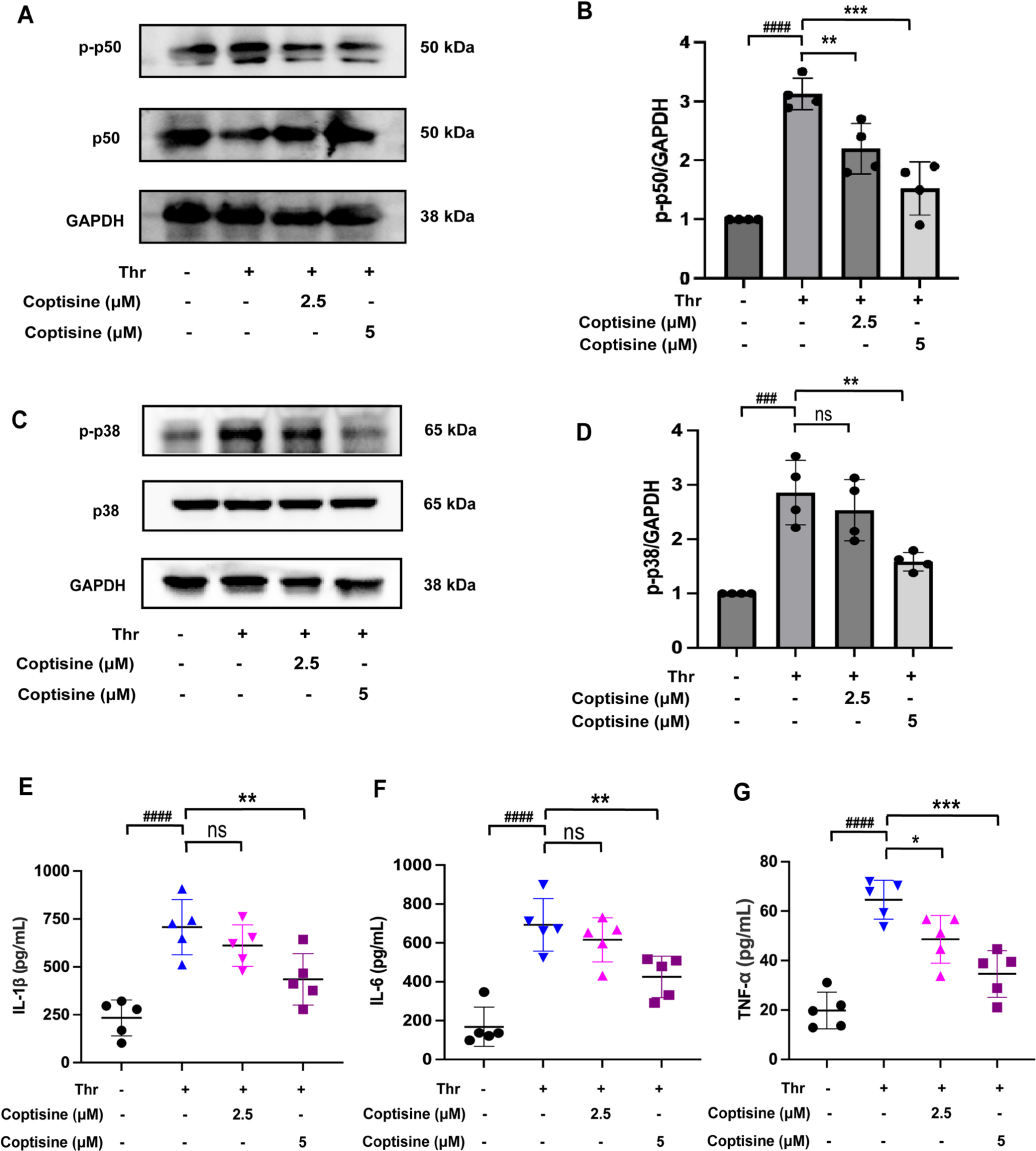
Effects of Coptisine on thrombin‐induced NF‐κB and MAPK signaling activation. (A) Platelets were pretreated with Coptisine at room temperature for 30 min, followed by stimulation with thrombin (0.025 U/mL). Representative Western blot images for phosphorylated P50 and total P50 (C) Representative Western blot images for phosphorylated P38 and total P38 (B, D). Histogram analysis of the immunoblots. Data are expressed as mean ± standard deviation. *n* = 4. ###*p* < 0.001 versus the control group, *p < 0.05, **p < 0.01, ***p < 0.001 versus the model group. (E, F, G) Levels of IL‐6, TNF‐α, and IL‐1β in mouse platelets were detected by enzyme‐linked immunosorbent assay (ELISA). All data are expressed as mean ± SD, *n* = 5, ###*p* < 0.001 versus the control group, ns = *p* > 0.05, *p < 0.05, **p < 0.01, ***p < 0.001 versus the model group.

Network pharmacology and molecular docking identified NFKB1 as a key target of coptisine via the NF‐κB pathway. Western blot confirmed that thrombin‐induced p‐P50 upregulation in platelets was suppressed by coptisine (Figure 6A,B).

Studies have shown that these inflammatory factors activate platelet membrane receptors in an autocrine manner, subsequently initiating p38 MAPK phosphorylation. The activation of p38 MAPK in platelets can further enhance IL‐1β release, creating an amplified cascade effect (Mao et al. 2025; Zhong et al. 2024). Therefore, we examined p‐P38 protein levels in platelets by WB and found that p‐P38 expression was upregulated in the thrombin‐induced platelet model, while coptisine treatment reversed this phenomenon (Figure 6C,D).

To determine whether coptisine could reduce inflammatory cytokine levels in thrombin‐induced platelets, we measured these cytokines using ELISA. The thrombin‐stimulated group showed significantly increased levels of TNF‐α, IL‐6, and IL‐1β, while coptisine treatment reduced their production (Figure 6E–G). These results demonstrate that coptisine can effectively inhibit both thrombin‐induced platelet activation and inflammatory responses through the NF‐κB/p38‐MAPK pathway.

### 3.7 Coptisine Inhibits Thrombus Formation but Has no Significant Impact on the Coagulation System

**FIGURE 7:**
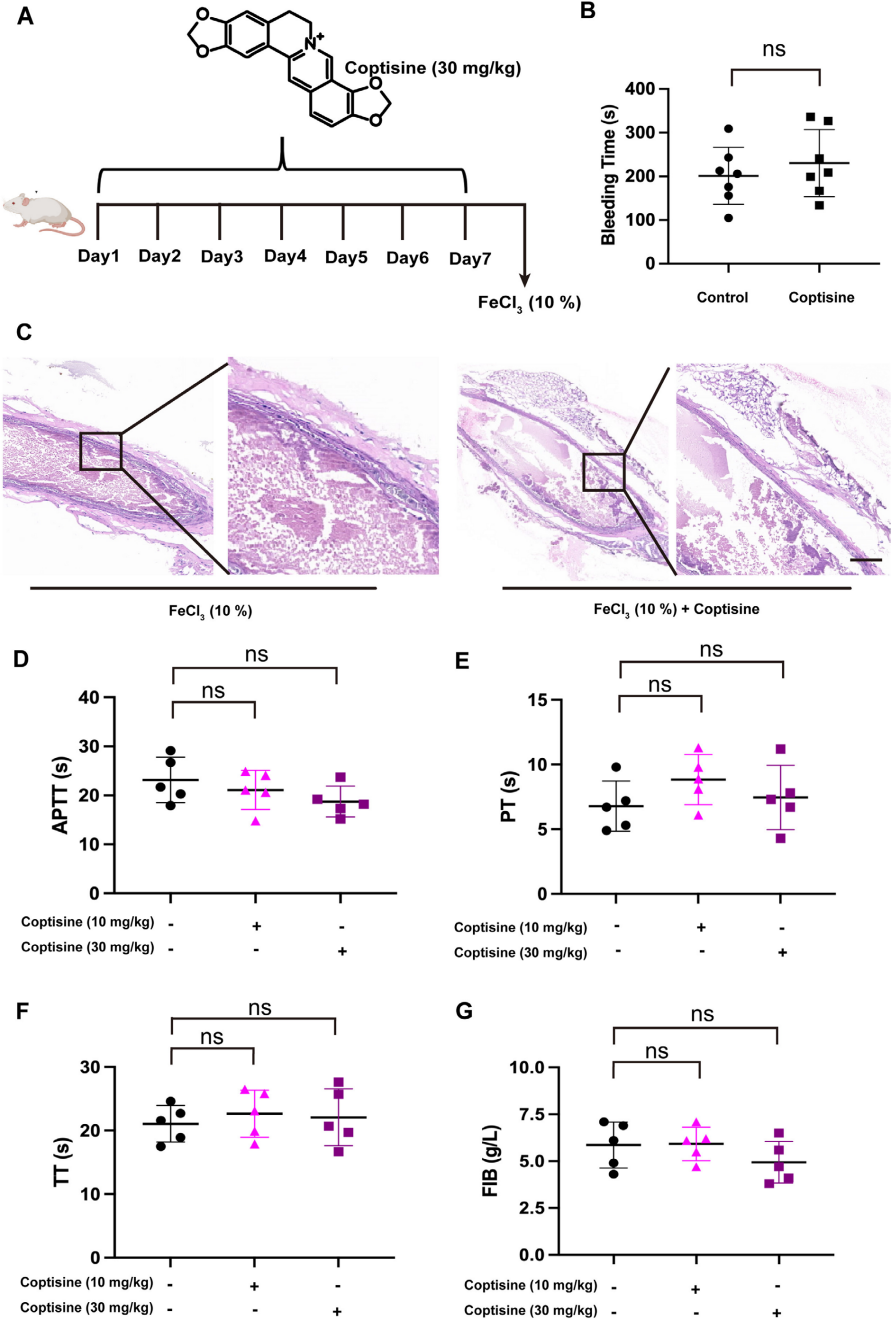
Coptisine inhibits thrombus formation but has no significant impact on the coagulation system. (A) In vivo experimental design. (B) Mice were orally administered coptisine (30 mg/kg) or vehicle control. The distal 2 mm of the tail was surgically excised and immediately immersed in pre‐warmed 0.9% NaCl solution, and the bleeding cessation time was recorded. *n* = 7, ns = *p* > 0.05. (C) H&E staining images of carotid artery thrombi (*n* = 3). (D–G) Effects of coptisine treatment on coagulation function indicators in rats: Prothrombin time (PT), activated partial thromboplastin time (APTT), thrombin time (TT), and fibrinogen (FIB) levels. Results are expressed as mean ± SD. *n* = 5, ns = *p* > 0.05. Scale bar represents 100 μm.

Next, we evaluated the effects of coptisine on in vivo thrombus formation and the coagulation system. The in vivo experimental design is summarized in Figure 7A. As shown in Figure 7B, coptisine did not significantly prolong tail bleeding time compared with the vehicle‐treated mice. In the FeCl_3_‐induced arterial thrombosis model, the histopathological examination of thrombus tissues showed that the thrombus structure in mice treated with coptisine was significantly less dense compared to that in the model group (Figure 7C). To validate the antithrombotic efficacy of coptisine, aspirin was included as a positive control in the supplementary experiments. The results demonstrated that coptisine exerted comparable effects to aspirin in ameliorating thrombus structure in mice (Figure S1). We further investigated the effects of coptisine on coagulation factors (Xin et al. 2017). The results showed that coptisine treatment did not significantly affect the activated partial thromboplastin time (APTT) (Figure 7D), prothrombin time (PT) (Figure 7E), thrombin time (TT) (Figure 7F), or fibrinogen (FIB) levels (Figure 7G). These findings suggest that coptisine may prevent venous and arterial thrombosis by inhibiting platelet activation.

## 4 Discussion

In recent years, natural medicines have garnered widespread attention in antiplatelet and antithrombotic therapies due to their multi‐target and low‐toxicity characteristics. Coptisine, a major active component of the traditional Chinese medicine Coptis chinensis, has been proven to possess various pharmacological effects, including anti‐inflammatory, antioxidant, and metabolic regulation properties (Li, Deng, et al. 2024; Tan et al. 2025a). However, the specific mechanisms by which Coptisine affects platelet activation and thrombus formation have not been fully elucidated. This study, for the first time, combines network pharmacology predictions with experimental validation to demonstrate that Coptisine significantly inhibits platelet activation and thrombus formation by regulating the NF‐κB and p38 MAPK signaling pathways, providing new molecular mechanistic evidence for its antithrombotic effects.

Platelet activation is a core component of thrombus formation, involving complex signal network regulation. NF‐κB, as a key transcription factor in inflammation and immune responses, has been found to play an important role in platelet activation in recent studies (Jiang et al. 2025). The activation of NF‐κB can promote the expression of P‐selectin in platelets and the release of pro‐inflammatory factors, thereby enhancing platelet aggregation and adhesion (Huang et al. 2022). Our experimental results show that Coptisine significantly inhibits the activation of the NF‐κB pathway in platelets induced by agonists and reduces the release of downstream inflammatory factors (such as TNF‐α and IL‐1β), indicating that Coptisine may attenuate platelet hyperreactivity by inhibiting the NF‐κB‐mediated inflammatory signaling pathway. Additionally, p38MAPK, an important member of the mitogen‐activated protein kinase (MAPK) family, plays a key regulatory role in platelet activation and thrombus formation (Tang et al. 2023). The activation of p38 MAPK can promote platelet cytoskeletal reorganization, granule release, and activation of integrin αIIbβ3, thereby enhancing platelet aggregation and thrombus stability (Guo et al. 2025).

Notably, in macrophages, genes that depend on both p38 and NF‐κB signaling (AND‐gate genes) exhibit high heterogeneity at the single‐cell level, systematically revealing the dynamic encoding and cooperative regulatory mechanisms of the p38 and NF‐κB signaling pathways (Luecke et al. 2024). A key study by Kaur et al. (2003) demonstrated that in thrombin‐stimulated endothelial cells, p38 MAPK activation is required for NF‐κB mobilization to the nucleus and subsequent E‐selectin‐dependent leukocyte recruitment, establishing p38 MAPK as a critical upstream regulator of NF‐κB‐dependent inflammatory responses. More directly relevant to platelets, a study utilizing the SARS‐CoV‐2 envelope protein showed that the p38 MAPK inhibitor SB203580 suppressed both p38 and NF‐κB phosphorylation in human platelets, indicating that p38 MAPK lies upstream of NF‐κB in platelet signaling cascades (Tang et al. 2023). Complementary evidence from Haematologica revealed that in thrombin‐activated human platelets, the nSMase/ceramide axis downstream of PAR4 selectively activates a p38 MAPK/NF‐κB cascade, with p38 MAPK functioning upstream of IKKβ phosphorylation and subsequent NF‐κB activation (Chen et al. 2013). Furthermore, platelets themselves serve as reservoirs of key inflammatory transcription regulators, including NF‐κB and p38 MAPK, and can transfer these regulators to other cell types via vesicle‐mediated mechanisms, highlighting the functional relevance of these pathways beyond nucleated cells (Hawwari et al. 2024). Collectively, these findings suggest the existence of a directional p38 MAPK/NF‐κB signaling axis in platelets.

Under thrombin stimulation, NF‐κB pathway activation may not only directly promote platelet activation but also induce the release of pro‐inflammatory factors such as TNF‐α and IL‐1β via autocrine/paracrine mechanisms. Additionally, p38 MAPK signaling interacts with the inflammasome pathway, a key mediator of pro‐inflammatory cytokine production, supporting the existence of a positive feedback loop (Liu, Nong, et al. 2023; Vervaeke and Lamkanfi 2025). These findings reveal a key link between inflammatory signaling and platelet activation. However, direct evidence for the NF‐κB/p38 feedback loop in platelets is lacking and warrants future investigation using inhibitors or genetic approaches.

Compared with traditional antiplatelet drugs (such as aspirin and clopidogrel), Coptisine has the advantage of multi‐target regulation. Aspirin mainly irreversibly inhibits COX‐1 to reduce the production of thromboxane A2 (TXA2) (Liani et al. 2026), while clopidogrel inhibits ADP‐mediated platelet activation by antagonizing the P2Y12 receptor (Tunstromer et al. 2023). However, long‐term use of these drugs may increase the risk of bleeding, and some patients exhibit “clopidogrel resistance” (Xie et al. 2025). This study shows that coptisine inhibits platelet activation in mice without affecting coagulation function or prolonging bleeding time, offering a favorable safety profile compared to traditional antiplatelet agents such as aspirin and clopidogrel. Mechanistically, upon thrombin stimulation, coptisine concurrently suppresses the NF‐κB and p38 MAPK signaling pathways, thereby restraining excessive platelet activation. These findings support the clinical potential of coptisine. Future research should focus on dose optimization and synergistic effects with other antiplatelet agents, with the aim of developing safer and more effective antithrombotic therapeutic strategies.

### 4.1 Limitations

This study reveals a novel mechanism by which coptisine inhibits platelet activation and thrombosis via NF‐κB and p38 MAPK pathways, but several limitations exist. Mechanistic evidence is primarily based on network pharmacology and in vitro experiments without causal validation using genetic approaches or specific inhibitors. Only male animals were used, limiting generalizability, and pharmacokinetic properties were not evaluated. Future studies will address these issues by incorporating genetic/pharmacological validation, including both sexes, and performing pharmacokinetic analyses to support clinical translation.

## 5 Conclusions

This study shows that coptisine dually modulates NF‐κB and p38 MAPK pathways to inhibit platelet activation and inflammation. Thrombin induces p‐P50 (NF‐κB) phosphorylation, triggering pro‐inflammatory factors (TNF‐α, IL‐1β) that activate p38 MAPK via autocrine/paracrine mechanisms, forming a positive “inflammation–activation” feedback loop. By suppressing both p‐P50 and p‐P38, coptisine blocks the NF‐κB inflammatory cascade and MAPK‐dependent granule secretion (e.g., ADP, 5‐HT), thereby coordinately inhibiting platelet hyperactivation and thrombus formation without bleeding risk‐a safer profile than traditional antiplatelet drugs. These findings support coptisine's antithrombotic application and open new avenues for multi‐target natural medicine strategies.

## Author Contributions

**Hui Yang:** investigation, writing – original draft. **Ming Qian:** conceptualization, data curation, writing – original draft, visualization. **Xian Yang:** supervision, writing – review and editing. **Xiaoli Zhou:** supervision, writing – review and editing. **Yanli Liu:** conceptualization, data curation, formal analysis, funding acquisition, writing – original draft, validation. **Yihai Liu:** funding acquisition, resources, writing – review and editing. **Shiyu Qian:** methodology, writing – review and editing. **Depin Li:** methodology, writing – review and editing.

## Funding

The authors declare financial support was received for the research, authorship, and/or publication of this article. This work was financed by Grant‐in‐aid for scientific research from the Youth Cultivation Program of the National Natural Science Foundation of China (NSFC), Nanjing Drum Tower Hospital (Grant No. 2024‐JCYJ‐QP‐75), the National Natural Science Foundation of China (82400399), National Science and Technology Major Project (2023ZD0514100), Key Project supported by Medical Science and technology development Foundation, Nanjing Department of Health (YKK24093), the Project of Institute of Chinese Medicine, Nanjing University (Grant No. ICM2024012), and the Project of State Key Laboratory of Functions and Applications of Medicinal Plants, Guizhou Medical University (FAMP20240X).

## Ethics Statement

The animal study was approved by Nanjing Drum Tower Hospital, Nanjing University. The study was conducted in accordance with the local legislation and institutional requirements.

## Conflicts of Interest

The authors declare no conflicts of interest.

## Supporting information


**Figure S1:** Coptisine protects mice against FeCl3‐induced thrombus formation. Representative H&E staining images of thrombi following FeCl_3_‐induced carotid artery injury are presented (*n* = 3). Wild‐type mice were pretreated with coptisine (30 mg/kg/day) for 7 days or aspirin (30 mg/kg/day) for 3 days, followed by FeCl_3_ injury. Scale bar represents 50 μm (left). Bar represents 10 μm (right).

## Data Availability

The raw data supporting the conclusions of this article will be made available by the authors, without undue reservation.
