# Supplementary material for: Exploring the Distinct Regulatory Actions and Molecular Pathways of Coptisine Through Network Pharmacology: Insights Into Its Influence on Platelet Activation and Thrombus Development
Source: Food Sci Nutr. 2026 Jul 15;14(7):e72107. doi: 10.1002/fsn3.72107 (PMC13370792; doi:10.1002/fsn3.72107)
Supplement: Supplementary file 1 — Figure S1: Coptisine protects mice against FeCl3‐induced thrombus formation. Representative H&E staining images of thrombi following FeCl3‐induced carotid artery injury are presented (n = 3). Wild‐type mice were pretreated with coptisine (30 mg/kg/day) for 7 days or aspirin (30 mg/kg/day) for 3 days, followed by FeCl3 injury. Scale bar represents 50 μm (left). Bar represents 10 μm (right). [file FSN3-14-e72107-s001.docx]

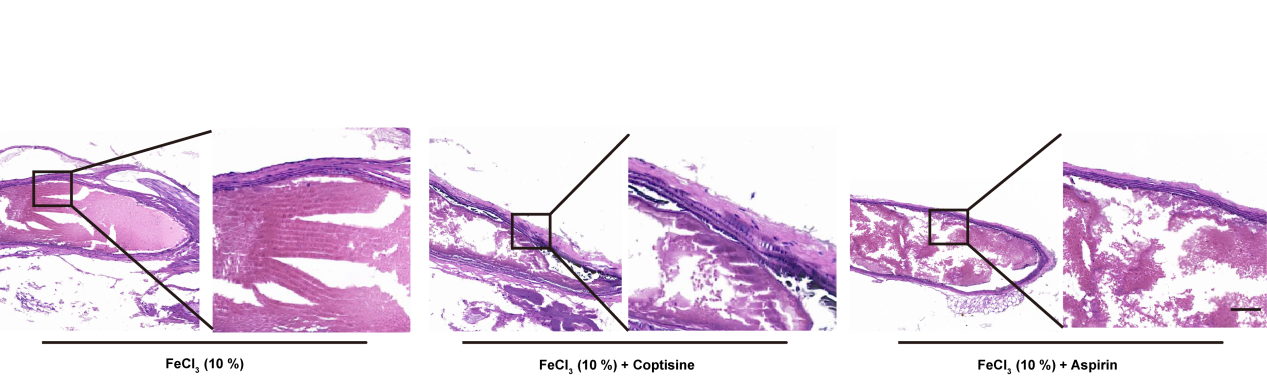


**Figure S1. Coptisine protects mice against FeCl_3_-induced thrombus formation.**  Representative H&E staining images of thrombi following FeCl_3_‑induced carotid artery injury are presented (n = 3). Wild-type mice were pretreated with coptisine (30 mg·kg⁻¹·d⁻¹) for 7 days or aspirin (30 mg·kg⁻¹·d⁻¹) for 3 days, followed by FeCl_3_ injury. Scale bar represents 50 μm (left). Bar represents 10 μm (right).
